# Supplementary material for: Structural basis of biofilm formation mediated by the Pseudomonas aeruginosa fibrillar adhesin CdrA
Source: bioRxiv. 2026 Jul 13:2026.07.13.738186. Preprint. [Version 1] doi: 10.64898/2026.07.13.738186 (PMC13405001; doi:10.64898/2026.07.13.738186)
Supplement: Supplementary file 1 [file NIHPP2026.07.13.738186v1-supplement-3.pdf]

bioRxiv preprint doi: https://doi.org/10.64898/2026.07.13.738186; this version posted July 13, 2026. The copyright holder for this preprint (which was not certified by peer review) is the author/funder, who has granted bioRxiv a license to display the preprint in perpetuity. It is made available under a CC-BY 4.0 International license.

Supplementary figures

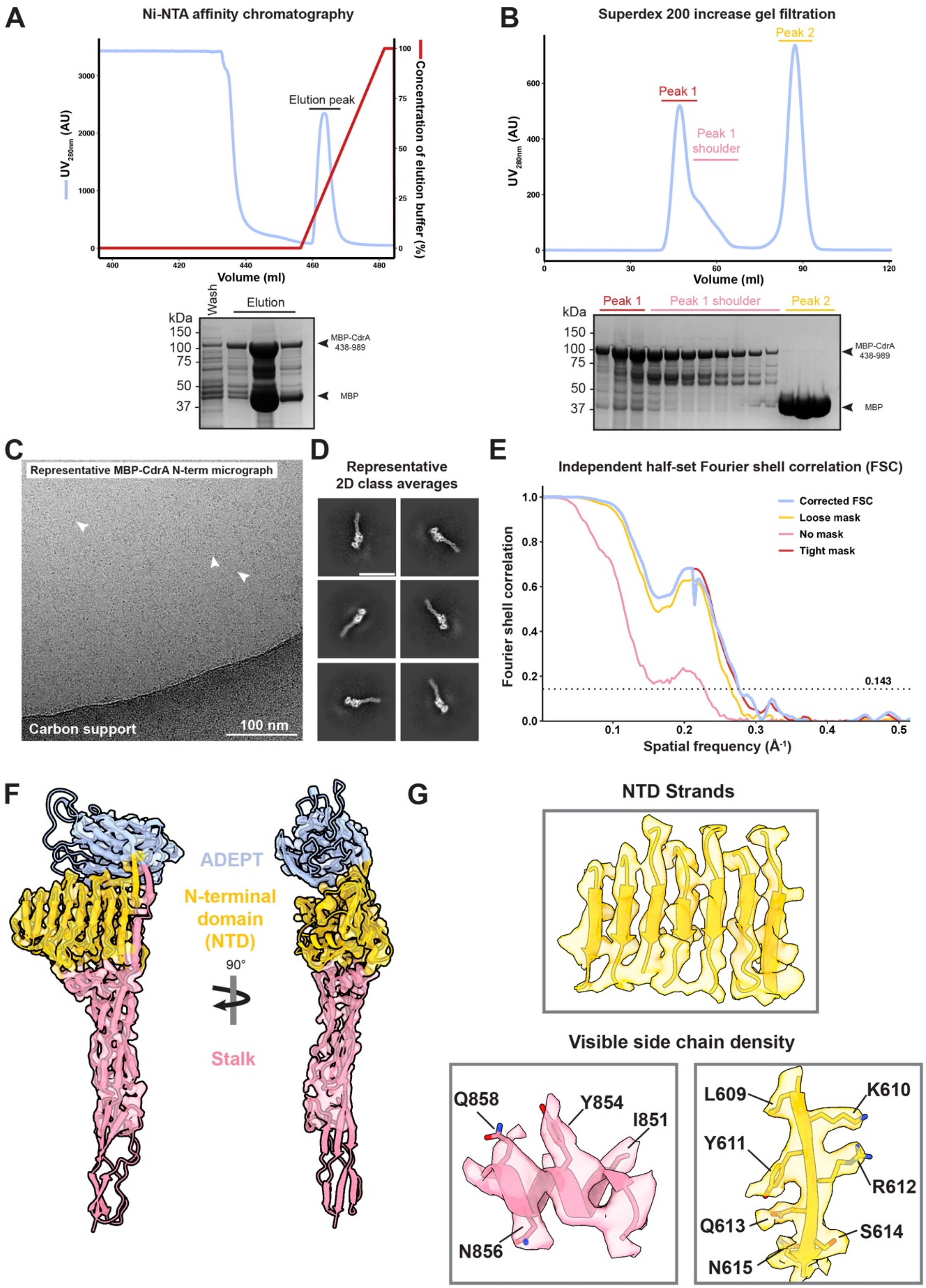

bioRxiv preprint doi: https://doi.org/10.64898/2026.07.13.738186; this version posted July 13, 2026. The copyright holder for this preprint (which was not certified by peer review) is the author/funder, who has granted bioRxiv a license to display the preprint in perpetuity. It is made available under a CC-BY 4.0 International license.

Fig. S1. Single particle cryo-EM structure of the CdrA adhesive N-terminus.

A) Nickel immobilised metal ion affinity chromatography of MBP-CdrA-438-989 construct. Chromatogram (top) displays a large UV peak in the elution which corresponded to the MBP-CdrA-438-989 construct when analysed by SDS-PAGE (bottom). Molecular weight standards are indicated on the left. The construct was further verified by peptide fingerprinting mass spectrometry. **B)** Gel filtration of the affinity purified MBP-CdrA-438-989 yielded a series of peaks in the UV trace (top). SDS-PAGE analysis (bottom) shows that peak 1 shoulder corresponds to MBP-CdrA-438-989 and was used for downstream work. Peak1 corresponds to soluble aggregates due to their proximity to the void volume. Peak 2 corresponds to free MBP. **C)** Representative micrograph of grids prepared with purified MBP-CdrA-438-989. White arrow heads point to representative particles. **D)** Representative 2D class averages obtained from averaging the merged single particle cryo-EM datasets of MBP-CdrA-438-989 and CdrA-438-989 (MBP-cleaved version). Secondary structure features are apparent in all parts of the protein. Scale bar = 150 Å. **E)** Fourier shell correlation (FSC - blue line) as calculated by cryoSPARC for the local refinement job which was used to produce the density for model building. The 0.143 criterion used for resolution estimation is shown as a dotted line. Masked curves are shown in different colours. Resolution of map = 3.62 Å. **F)** Fit of CdrA adhesive N-terminus model into the cryo-EM map. The mask used in local refinement excluded the terminal repeat hence the absence of strong density for this region. Domains are coloured, with ADEPT = blue, NTD = yellow, stalk = pink. **G)** Zoom in of different parts of the model into map fit. Top: fit of the model in the sheet region in the NTD. Residues shown in cartoon schematic (from left to right β-strands: 439-445, 484-496, 512-519, 534-541, 565-572, 586-594, 606-614). Bottom left: side chain density for residues in the stalk helix: I851, Y854, N856 and Q858. Bottom right: side chain density for residues in one of the NTD strands: L609, K610, Y611, R612, Q613, S614, N615. Residue numbers refer to residues in full-length CdrA. Side chains are labelled by heteroatom (with

bioRxiv preprint doi: https://doi.org/10.64898/2026.07.13.738186; this version posted July 13, 2026. The copyright holder for this preprint (which was not certified by peer review) is the author/funder, who has granted bioRxiv a license to display the preprint in perpetuity. It is made available under a CC-BY 4.0 International license.

nitrogen atoms represented in blue and oxygen atoms in red) and hydrogen atoms have been hidden.

bioRxiv preprint doi: https://doi.org/10.64898/2026.07.13.738186; this version posted July 13, 2026. The copyright holder for this preprint (which was not certified by peer review) is the author/funder, who has granted bioRxiv a license to display the preprint in perpetuity. It is made available under a CC-BY 4.0 International license.

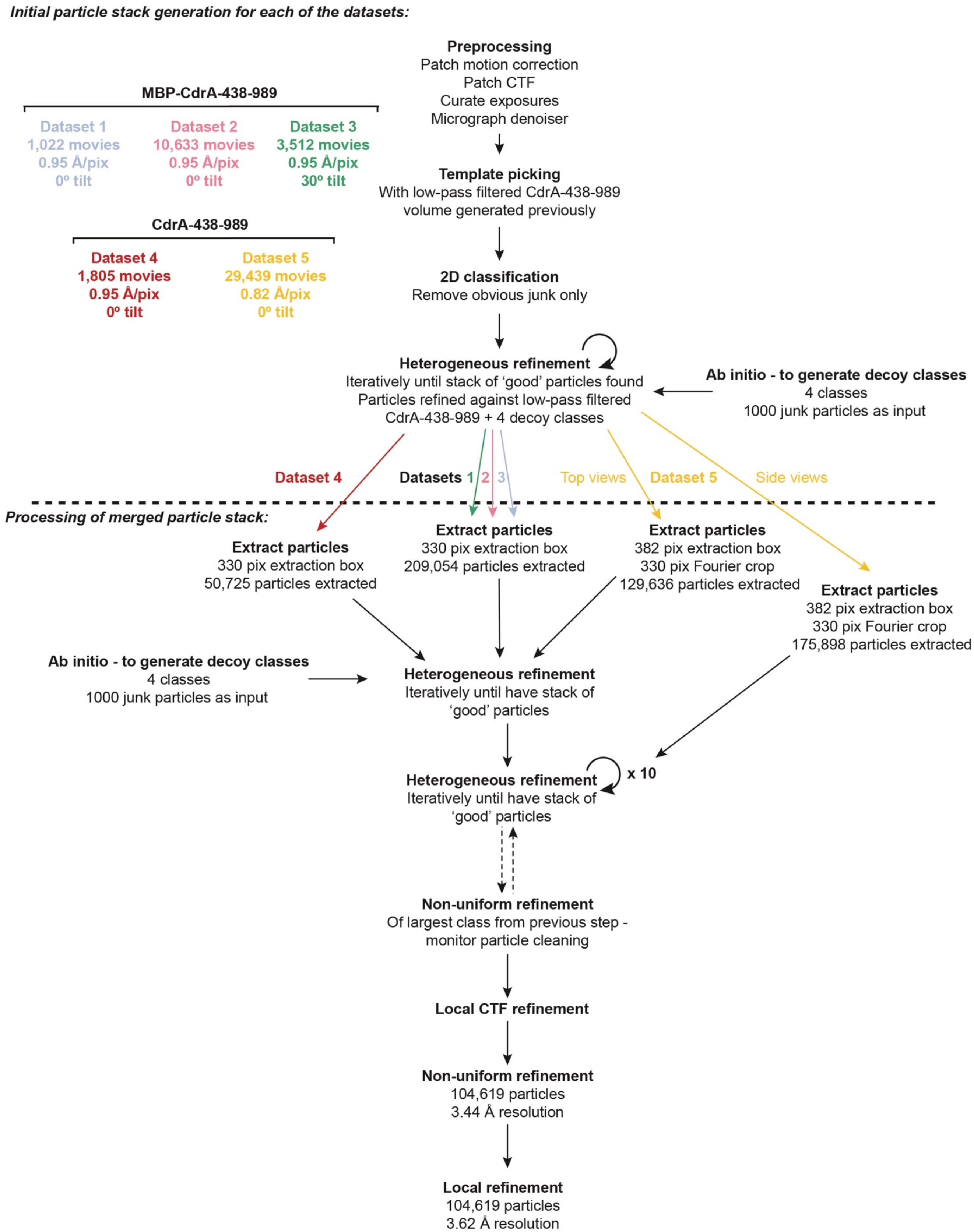

bioRxiv preprint doi: https://doi.org/10.64898/2026.07.13.738186; this version posted July 13, 2026. The copyright holder for this preprint (which was not certified by peer review) is the author/funder, who has granted bioRxiv a license to display the preprint in perpetuity. It is made available under a CC-BY 4.0 International license.

Fig. S2. Simplified single particle cryo-EM workflow for CdrA adhesive N-terminus structure determination.

Data processing workflow of individual datasets (top) to produce a stack of particles which was merged (bottom) and particles processed together further to yield a volume at 3.62 Å resolution (as calculated by cryoSPARC using the 0.143 GS-FSC criterion). All processing steps were carried out using cryoSPARC v4.5.1.

bioRxiv preprint doi: https://doi.org/10.64898/2026.07.13.738186; this version posted July 13, 2026. The copyright holder for this preprint (which was not certified by peer review) is the author/funder, who has granted bioRxiv a license to display the preprint in perpetuity. It is made available under a CC-BY 4.0 International license.

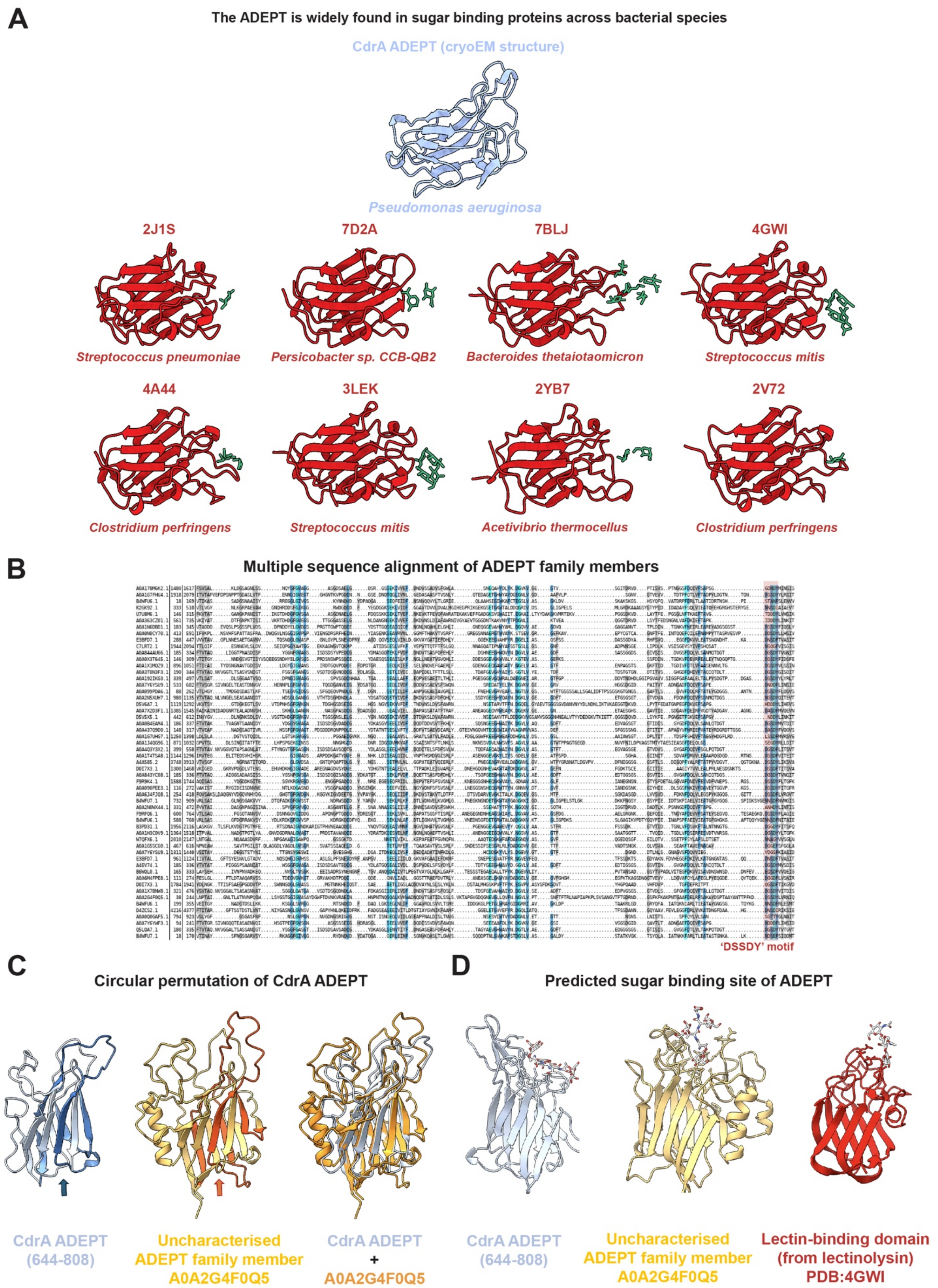

bioRxiv preprint doi: https://doi.org/10.64898/2026.07.13.738186; this version posted July 13, 2026. The copyright holder for this preprint (which was not certified by peer review) is the author/funder, who has granted bioRxiv a license to display the preprint in perpetuity. It is made available under a CC-BY 4.0 International license.

Fig. S3. Circular permutation in CdrA ADEPT and similarity of its predicted sugar binding site to known adhesive domains.

A) Cryo-EM structure of the CdrA ADEPT (residues 644 – 808) with the experimental structures of other structurally-similar domains, members of the galactose-binding domain superfamily (coloured red; Protein Data Bank (PDB) codes provided) with their co-crystallised sugar ligands (green). **B)** Multiple sequence alignment of ADEPT family members. Colours in MSA represent conservation of residues and proteins are listed with their Uniprot codes. The location of the conserved 'DSSDY' motif is highlighted in red. **C)** Comparison of the AlphaFold2 (AF2) models of the CdrA ADEPT (residues 644 – 808, shown in blue) and an uncharacterised member of ADEPT family (UniprotKB:A0A2G4F0Q5, residues 1 – 250, shown in orange). The circularly permuted region, which is located at the N-terminus of CdrA ADEPT and at the C-terminus of A0A2G4F0Q5 ADEPT, is shown in dark blue and red-orange, respectively (with coloured arrows to indicate this). **D)** Side-by-side comparison of the AlphaFold3 (AF3) predicted models of CdrA (shown in blue) and A0A2G4F0Q5 (shown in orange) bound to peptide sugar mimetic; and the crystal structure of the lectin binding domain of lectinolysin (shown in red) in complex with Lewis y (PDB:4GWI).

bioRxiv preprint doi: https://doi.org/10.64898/2026.07.13.738186; this version posted July 13, 2026. The copyright holder for this preprint (which was not certified by peer review) is the author/funder, who has granted bioRxiv a license to display the preprint in perpetuity. It is made available under a CC-BY 4.0 International license.

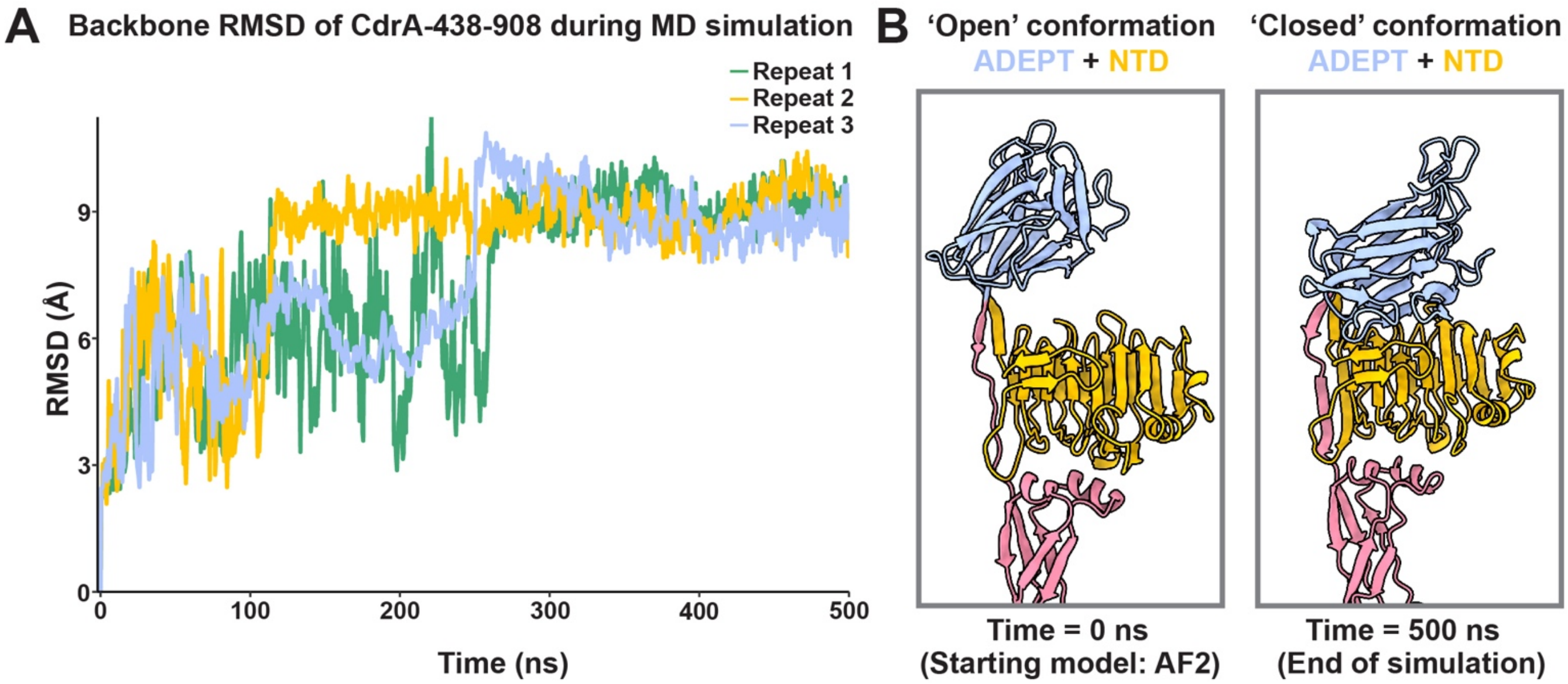

Fig. S4. Molecular dynamics simulations of the CdrA N-terminus (residues 438-908).

A) Plot displaying CdrA backbone root mean square deviation (RMSD) changes as molecular dynamics (MD) simulation proceeds. The AlphaFold2 model of the CdrA N-terminus (from residues 438-908 - including NTD, ADEPT and one stalk repeat) was allowed to explore RMSD changes in a solvent box for a total simulation time of 500 ns. The simulation was repeated 3 times, with repeats indicated by differentially coloured lines: repeat 1 = green, repeat 2 = yellow & repeat 3 = blue. **B)** Conformations of CdrA-438-989 at the beginning of the simulation (0 ns) and at the end (500 ns). CdrA-438-989 is coloured by domain, with: NTD = yellow, ADEPT = blue, stalk repeat = pink. At the beginning of the simulation, a much larger distance in the ADEPT:NTD interface is observed with a more 'open' conformation. At the end of the simulation, a much closer distance in the ADEPT:NTD interface is observed, with CdrA-438-989 in a 'closed' conformation.

bioRxiv preprint doi: https://doi.org/10.64898/2026.07.13.738186; this version posted July 13, 2026. The copyright holder for this preprint (which was not certified by peer review) is the author/funder, who has granted bioRxiv a license to display the preprint in perpetuity. It is made available under a CC-BY 4.0 International license.

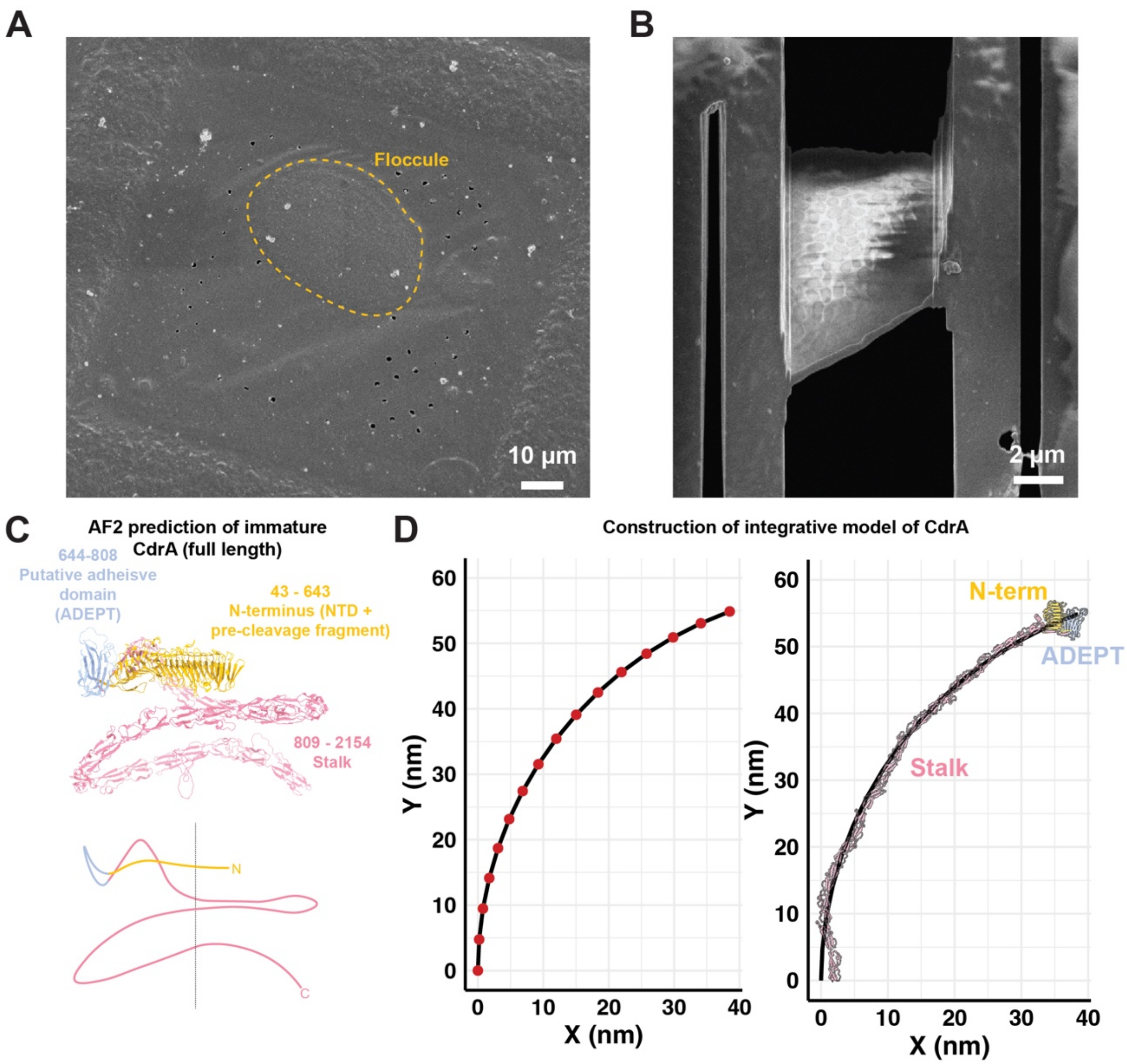

Fig. S5. Calculation of the *in situ* model of CdrA.

A) SEM image of a CdrA-mediated floccule frozen on an electron microscopy grid prior to focused-ion beam (FIB) milling. Region highlighted in yellow corresponds to a CdrA-mediated floccule and was targeted for FIB milling. **B)** SEM image of lamella of floccule produced from FIB milling. **C)** AlphaFold3 prediction of full length CdrA in a condensed, coiled-up architecture. Domain boundaries for full-length CdrA are indicated and the prediction is coloured by domain: N-terminus (NTD + pre-cleavage fragment (residues 43-437)), ADEPT and stalk. **D)** Left hand plot displays an estimation for the curvature of CdrA which was used for production of the *in situ* integrative model of CdrA. The coordinates were used as restraints for molecular dynamics simulation.

bioRxiv preprint doi: https://doi.org/10.64898/2026.07.13.738186; this version posted July 13, 2026. The copyright holder for this preprint (which was not certified by peer review) is the author/funder, who has granted bioRxiv a license to display the preprint in perpetuity. It is made available under a CC-BY 4.0 International license.

Coordinates were derived from fitting the average length of CdrA (69nm) with the measured radius of curvature of CdrA (58 nm; with the assumption that curvature is constant throughout the CdrA molecule). On the right, the integrative model of CdrA is overlaid onto these points, displaying the fit of the model to the coordinates after molecular dynamics simulations.

bioRxiv preprint doi: https://doi.org/10.64898/2026.07.13.738186; this version posted July 13, 2026. The copyright holder for this preprint (which was not certified by peer review) is the author/funder, who has granted bioRxiv a license to display the preprint in perpetuity. It is made available under a CC-BY 4.0 International license.

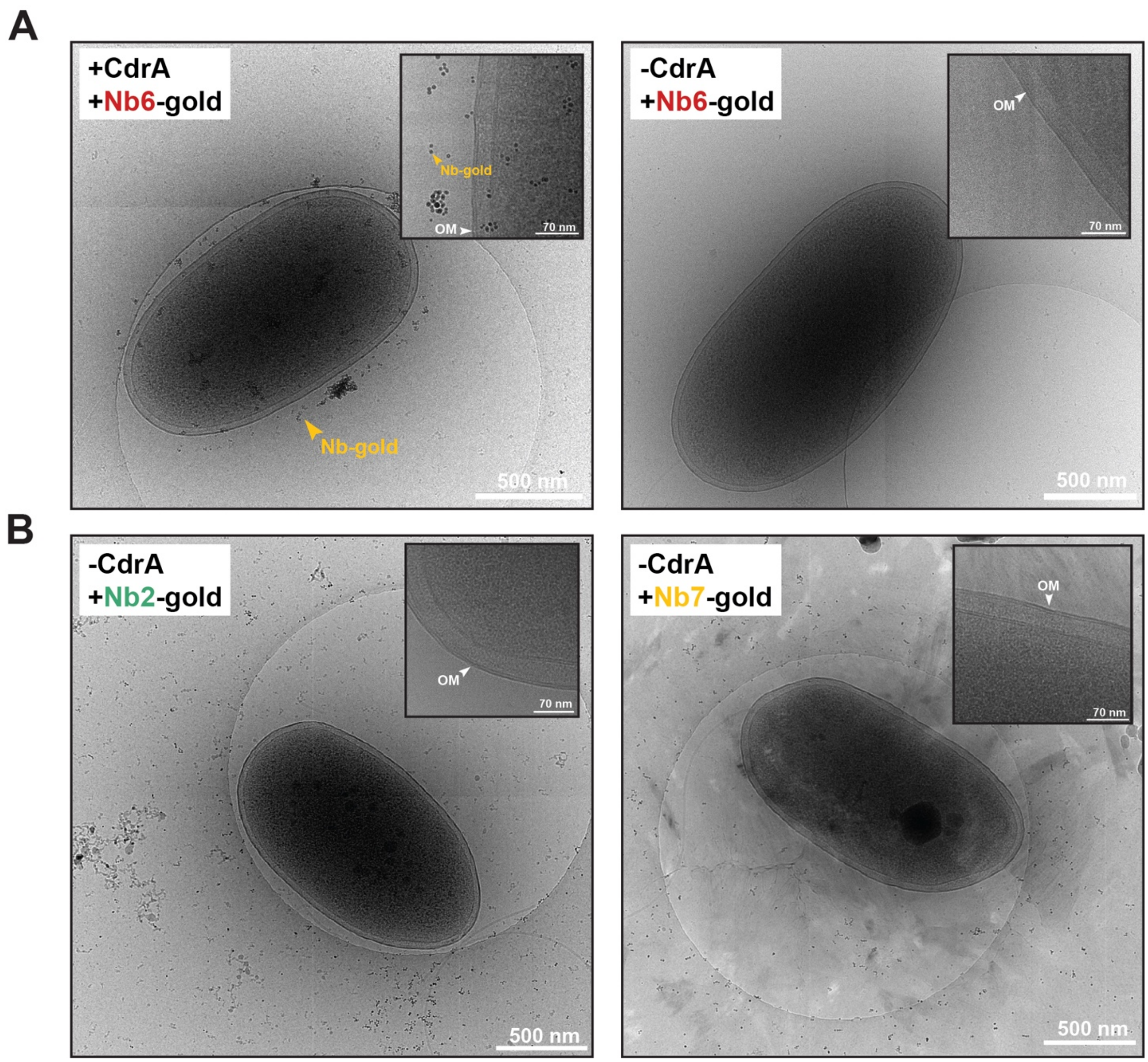

Fig. S6. Anti-CdrA nanobodies specifically label CdrA at the cell-surface.

A) Cryo-EM images of cells labelled with Nb6-gold. Left panel shows a Nb6-gold labelled cell where CdrA is expressed (hence high levels of surface Nb6-gold decoration). The zoomed inset (left) shows that the cell-surface is highly decorated with Nb6-gold. The right image shows an unexpressed CdrA control where no CdrA is expressed and as a result, there is no Nb6-gold labelling at the cell-surface. The zoomed inset (right) also displays an absence of Nb6-gold labelling at the cell-surface. OM = outer membrane. For all images, the scale bar in the main panel represents 500 nm and scale bar in the inset represents 70 nm. **B)** Cryo-EM images of

bioRxiv preprint doi: https://doi.org/10.64898/2026.07.13.738186; this version posted July 13, 2026. The copyright holder for this preprint (which was not certified by peer review) is the author/funder, who has granted bioRxiv a license to display the preprint in perpetuity. It is made available under a CC-BY 4.0 International license.

CdrA controls for Nb2-gold (left hand side) and Nb7-gold (right hand side) on cells with no CdrA expression. Scale bars = 500 nm. For both, there is also no cell-surface decoration of Nb-gold (see insets). In the right-hand main image for Nb7-gold, the features around the cell are freezing artefacts due to crystalline ice. For all images, the scale bar in main panel represents 500 nm and the scale bar in inset represents 70 nm.

bioRxiv preprint doi: https://doi.org/10.64898/2026.07.13.738186; this version posted July 13, 2026. The copyright holder for this preprint (which was not certified by peer review) is the author/funder, who has granted bioRxiv a license to display the preprint in perpetuity. It is made available under a CC-BY 4.0 International license.

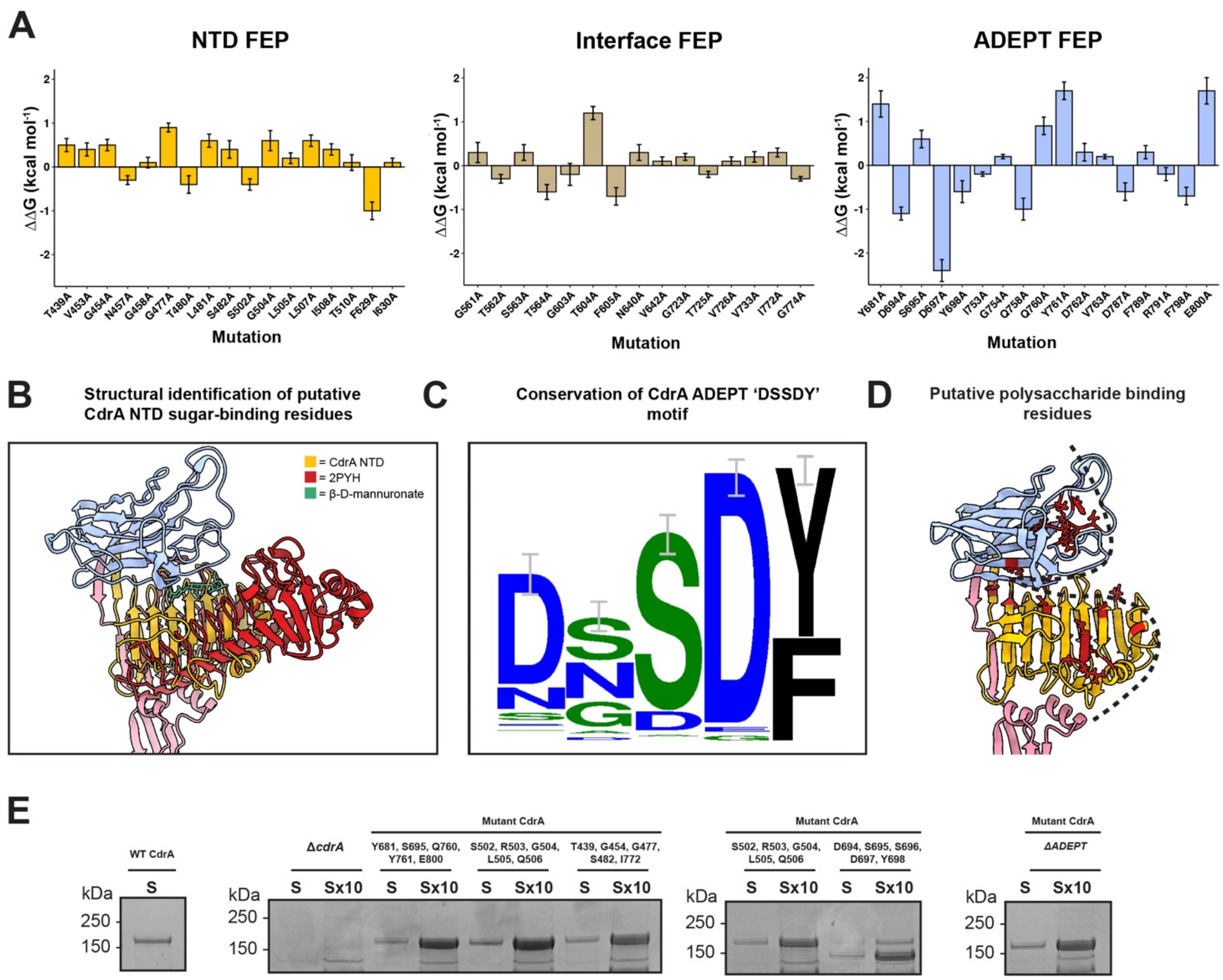

Fig. S7. Mutations to CdrA to probe sugar binding.

A) Free energy perturbation (FEP) plots displaying the change in free energy (ΔΔG) when the specified residues were mutated to alanine. A positive ΔΔG value suggests that mutation to alanine decreases the affinity to Psl compared to the WT. Plots shown are for residues in the NTD (yellow), the ADEPT:NTD interface (beige) and the ADEPT (blue). **B)** Superposition of the cryoEM structure (from this study) of the CdrA NTD (yellow) with a crystal structure of the right-handed β-helix domain of mannuronan C-5 epimerase AlgE4 - PDB code: 2PYH (red). The position of the co-crystallised sugar β-D-mannuronate (green) indicated putative sugar binding residues in the NTD that were mutated. **C)** Sequence alignment of the 'DSSDY' motif in the loop region of the CdrA ADEPT shows a high level of sequence conservation. Error bars represent the approximate

bioRxiv preprint doi: https://doi.org/10.64898/2026.07.13.738186; this version posted July 13, 2026. The copyright holder for this preprint (which was not certified by peer review) is the author/funder, who has granted bioRxiv a license to display the preprint in perpetuity. It is made available under a CC-BY 4.0 International license.

Bayesian 95% confidence interval. The 'DSSDY' motif logo shown here was produced using Weblogo (Crooks *et al.*, 2004). **D)** Mapping of all the experimentally mutated residues to the CdrA adhesive N-terminus reveals a putative polysaccharide binding pocket (indicated by dashed black line). **E)** SDS-PAGE analysis of sheared protein from the surface of cells overexpressing mutant CdrA constructs. Molecular weight standards are indicated on the left. CdrA expected molecular weight is ~176 kDa. CdrA Δ*ADEPT* expected molecular weight is 156 kDa. S = total sheared protein; Sx10 = total sheared protein 10x concentrated.

bioRxiv preprint doi: https://doi.org/10.64898/2026.07.13.738186; this version posted July 13, 2026. The copyright holder for this preprint (which was not certified by peer review) is the author/funder, who has granted bioRxiv a license to display the preprint in perpetuity. It is made available under a CC-BY 4.0 International license.

Tables

	MBP-CdrA-438-989			CdrA-438-989	
**Data collection**					
Microscope	Titan Krios	Titan Krios	Titan Krios	Titan Krios	Titan Krios
Detector	Falcon 4i (TFS)	Falcon 4i (TFS)	Falcon 4i (TFS)	Falcon 4i (TFS)	K3 (Gatan)
Software	EPU	EPU	EPU	EPU	EPU
Magnification	130,000 x	130,000 x	130,000 x	130,000 x	105,000 x
Voltage (kV)	300	300	300	300	300
Electron exposure (e^-/Å^2)	40	40	40	40	40
Slit width (eV)	20	20	20	20	20
Defocus range (µm)	-1.25 to -2.5	-1.25 to -2.5	-1.25 to -2.5	-1.25 to -2.5	-1.25 to 2.5
Pixel size (Å)	0.955	0.955	0.955	0.955	0.825
Stage tilt	0°	0°	30°	0°	0°
Movies collected	1,022	10,633	3,512	1,805	29,439
Movies used	870	9,576	2,310	1,596	25,266
Frames per movie	40	40	40	40	40
**Data processing**					
Software reconstruction	CryoSPARC v4.5.1	CryoSPARC v4.5.1	CryoSPARC v4.5.1	CryoSPARC v4.5.1	CryoSPARC v4.5.1
Initial number of particles extracted from micrographs (no.)		3,759,725		483,249	18,692,692
Input particles into merging all datasets		209,054		50,725	129,636 (top views)<br>175,898 (side views)
Final particle images after merging all datasets (no.)			104,619		
Final box size (px)	330 x 330 x 330	330 x 330 x 330	330 x 330 x 330	330 x 330 x 330	330 x 330 x 330
Pixel size final reconstruction (Å)	0.955	0.955	0.955	0.955	0.955
Symmetry imposed	-	-	-	-	-
Map resolution (Å)			3.62		
FSC threshold			0.143		
Map sharpening *B* factor (Å^2)			-72.4		
**Model refinement**					
Initial model used			AF3 Prediction of CdrA-438-989		
Software			Phenix		
Model resolution (Å) (FSC threshold -0/0.143/0.5)			3.3/3.6/4.0		
Model composition					
Non-hydrogen atoms			7918		
Protein residues			556		
B factors (Å^2)					
Protein			24.85		
RMS deviations					
Bond lengths (Å)			0.003		
Bond angles (°)			0.581		
Validation					
MolProbity score			1.86		
Clashscore			7.58		
Rotamer outliers (%)			0.00		
CABLAM outliers (%)			3.99		
Ramachandran plot					
Favoured (%)			93.14		
Allowed (%)			6.86		
Disallowed (%)			0.00		

Table S1 - Cryo-EM data collection, refinement, and validation statistics.

bioRxiv preprint doi: https://doi.org/10.64898/2026.07.13.738186; this version posted July 13, 2026. The copyright holder for this preprint (which was not certified by peer review) is the author/funder, who has granted bioRxiv a license to display the preprint in perpetuity. It is made available under a CC-BY 4.0 International license.

Mutation set	Targeting	Source of mutations
T439A, G454A, G477A, S482A, I772A	NTD	FEP simulations
S502A, R503A, G504A, L505A, E506A	NTD	Structural alignment with PDB:2PYH (same superfamily as CdrA NTD) and subsequent docking with Psl exopolysaccharide
Q561A, S563A, T604A, N640A, I772A	ADEPT:NTD	FEP simulations
Y681A, S695A, Q760A, Y761A, E800A	ADEPT	FEP simulations
D694A, S695A, S696A, D697A, Y698A	ADEPT	Comparison with other ADEPT family members – 'DSSDY' motif

Table S2. Mutations to the ADEPT, ADEPT:NTD interface and NTD.

For each penta-alanine mutation set, the domain or interface of CdrA that the mutation set targets is shown as well as the source for each mutation.

bioRxiv preprint doi: https://doi.org/10.64898/2026.07.13.738186; this version posted July 13, 2026. The copyright holder for this preprint (which was not certified by peer review) is the author/funder, who has granted bioRxiv a license to display the preprint in perpetuity. It is made available under a CC-BY 4.0 International license.

Movie Legends

Movie S1. Cryo-EM structure of the CdrA adhesive N-terminus.

A 3.6 Å resolution cryo-EM density map of the adhesive N-terminus is displayed. This density map was used to build an atomic model of the adhesive N-terminus (including the ADEPT, NTD and two stalk repeats). The map and model are coloured by domain (ADEPT – blue, NTD – yellow, stalk – pink) and both surface depiction and ribbon diagrams are shown.

Movie S2. *In situ* arrangement of CdrA.

A tomogram of a FIB-milled CdrA-dependent floccule is displayed which is segmented to display cell membranes (green) and the fibrillar CdrA molecules (pink).
